# Measuring the Outreach Efforts of Public Health Authorities and the Public Response on Facebook During the COVID-19 Pandemic in Early 2020: Cross-Country Comparison

**DOI:** 10.2196/19334

**Published:** 2020-05-19

**Authors:** Aravind Sesagiri Raamkumar, Soon Guan Tan, Hwee Lin Wee

**Affiliations:** 1 Saw Swee Hock School of Public Health National University of Singapore Singapore Singapore; 2 Department of Pharmacy National University of Singapore Singapore Singapore

**Keywords:** COVID-19, sentiment analysis, emotion analysis, public health authorities, infectious disease, outbreak, public engagement, social media, public health, virus

## Abstract

**Background:**

The coronavirus disease (COVID-19) pandemic presents one of the most challenging global crises at the dawn of a new decade. Public health authorities (PHAs) are increasingly adopting the use of social media such as Facebook to rapidly communicate and disseminate pandemic response measures to the public. Understanding of communication strategies across different PHAs and examining the public response on the social media landscapes can help improve practices for disseminating information to the public.

**Objective:**

This study aims to examine COVID-19-related outreach efforts of PHAs in Singapore, the United States, and England, and the corresponding public response to these outreach efforts on Facebook.

**Methods:**

Posts and comments from the Facebook pages of the Ministry of Health (MOH) in Singapore, the Centers for Disease Control and Prevention (CDC) in the United States, and Public Health England (PHE) in England were extracted from January 1, 2019, to March 18, 2020. Posts published before January 1, 2020, were categorized as pre-COVID-19, while the remaining posts were categorized as peri-COVID-19 posts. COVID-19-related posts were identified and classified into themes. Metrics used for measuring outreach and engagement were frequency, mean posts per day (PPD), mean reactions per post, mean shares per post, and mean comments per post. Responses to the COVID-19 posts were measured using frequency, mean sentiment polarity, positive to negative sentiments ratio (PNSR), and positive to negative emotions ratio (PNER). Toxicity in comments were identified and analyzed using frequency, mean likes per toxic comment, and mean replies per toxic comment. Trend analysis was performed to examine how the metrics varied with key events such as when COVID-19 was declared a pandemic.

**Results:**

The MOH published more COVID-19 posts (n=271; mean PPD 5.0) compared to the CDC (n=94; mean PPD 2.2) and PHE (n=45; mean PPD 1.4). The mean number of comments per COVID-19 post was highest for the CDC (mean CPP 255.3) compared to the MOH (mean CPP 15.6) and PHE (mean CPP 12.5). Six major themes were identified, with posts about prevention and safety measures and situation updates being prevalent across the three PHAs. The themes of the MOH’s posts were diverse, while the CDC and PHE posts focused on a few themes. Overall, response sentiments for the MOH posts (PNSR 0.94) were more favorable compared to response sentiments for the CDC (PNSR 0.57) and PHE (PNSR 0.55) posts. Toxic comments were rare (0.01%) across all PHAs.

**Conclusions:**

PHAs’ extent of Facebook use for outreach purposes during the COVID-19 pandemic varied among the three PHAs, highlighting the strategies and approaches that other PHAs can potentially adopt. Our study showed that social media analysis was capable of providing insights about the communication strategies of PHAs during disease outbreaks.

## Introduction

### Background

The coronavirus disease (COVID-19) was first identified in Wuhan, China in December 2019. It has since spread to 210 countries and territories, infecting 1,697,356 people and causing 102,667 deaths as of April 11, 2020 [1]. Compared to the previous outbreaks of severe acute respiratory syndrome (SARS) and Middle East respiratory syndrome, COVID-19 has caused more infections and deaths, spreading from an infected person to 2-2.5 people on average [2]. Most countries started reporting infections by the second half of January 2020. The United States reported its first case on January 20, 2020 [3], while England, under the United Kingdom, reported its first cases on January 31, 2020 [4]. Singapore reported its first case on January 23, 2020 [5]. In an effort to contain the COVID-19 pandemic in Singapore, multiple interventions have been implemented on both societal and health care system levels [6], and the country shifted rapidly to Disease Outbreak Response System Condition (DORSCON) orange, the second-highest level of alert for disease outbreaks in Singapore, on February 7, 2020 [7], just 15 days after the first case of COVID-19 infection was confirmed. The World Health Organization (WHO) declared this disease as a pandemic on March 11, 2020, and is unable to ascertain the duration of the pandemic [8].

Countries such as Singapore, Taiwan, and South Korea have taken the necessary precautions to handle this pandemic within their borders in January when the pandemic was largely confined to China [9]. On the international scene, Singapore has received accolades from the WHO and several world leaders praising our efforts in containing the disease [10,11]. The Dean of the Saw Swee Hock School of Public Health, National University of Singapore, has also been invited by several overseas universities to share Singapore’s experience in combating the COVID-19 pandemic [12,13]. Unfortunately, precautionary measures have been reported to be found wanting in other countries such as the United States and the United Kingdom [14,15]. It is to be noted that such delays in preparation for epidemics have also been seen in the past with Zika, influenza A virus subtype H1N1, and Ebola [16].

### Social Media Use During Pandemics

Effective risk communication is essential in directing the public to adopt certain desired behaviors such as social distancing and good hygiene habits in times of pandemics. Transparent and consistent communication amidst the uncertainty of the pandemic is also crucial in maintaining public confidence and trust [17,18]. Traditionally, the government and public health authorities (PHAs) relied on websites, news media, print press, and television as main platforms for the dissemination of pandemic-related news and information to the public. In contrast to the 2003 SARS and 2009 H1N1 pandemic, present-day media landscapes worldwide have evolved significantly, with a greater presence of social media and alternative local and overseas media outlets [19]. The advent of social media platforms such as Facebook and Twitter facilitated the instantaneous sharing of information during pandemics for both the health authorities and the general public. With widespread social media use and the participatory web, PHAs must understand that health risk communication is no longer a linear process [20]. The public can voice their sentiments and comments on the actions undertaken by the government as events related to the pandemic unfold. The public themselves are also involved in content creation through blogs and forums. Citizen journalism and propagation of information pertaining to a pandemic is made possible within their social networks.

Existing research on social media has explored epidemics and pandemics such as Zika [21-23], H1N1 [24], and Ebola [25]. The scope of these studies includes descriptive analysis of posting frequency [21], thematic analysis of post content [26], sentiment analysis of posts [23], and social network analysis [24]. Although the WHO has put forth guidelines for emergency risk communication during epidemics [27], countries may adopt different strategies when conveying health risks across social media platforms. Currently, there is a lack of studies that compare the social media outreach efforts of PHAs from different countries and corresponding responses and interactions by the general public. Such studies might offer rich insights on how effectively platforms such as Facebook could be used for risk communication.

### This Study

Amid the uncertainty of a health threat such as COVID-19, the public have a greater demand for real time, transparent, and consistent messaging. Government agencies run the risk of losing the centralized control of the risk communication process if they do not act swiftly to public sentiment and dispel falsehoods and misinformation [18,28]. A confluence of factors could lead to unintended behavioral outcomes among the public in the ongoing COVID-19 pandemic. The mismatch in perceived threats as well as costs and benefits of certain health behaviors communicated by either mainstream media, government authorities, or alternative media could result in a distorted understanding among the general public. Hence, it is crucial to understand how the prevailing sentiments and narratives about the pandemic were conveyed through the different communication channels and how it was received by the general public who have access to these channels. This will highlight the trigger points, allowing health authorities to fine-tune messaging along the course of the pandemic to allay public fear and panic.

Hence, in this study, we seek to answer four research questions related to Facebook use during a pandemic. First, how frequently do the PHAs of Singapore, the United States, and England use Facebook for risk communication? Second, what were the primary themes of the COVID-19-related posts by PHAs? Third, what are the Facebook followers’ sentiments and emotions in response to these COVID-19-related posts by PHAs? Fourth, how common are toxic comments that may incite public unrest, and do these toxic comments gain traction? We have selected Singapore, the United States, and England for this study, as we intend to look at the findings from a cross-country perspective and these are developed countries that have English as their official language.

## Methods

### Data Extraction

Data for this study were extracted from three Facebook pages using the tool Facepager [29] on March 19, 2020. The three Facebook pages are officially managed by the Ministry of Health (MOH), Singapore [30], the Centers for Disease Control and Prevention (CDC) in the United States [31], and Public Health England (PHE) in England [32]. As of April 4, 2020, the followers count of the MOH, the CDC, and PHE are 212,453, 2,636,072, and 336,935, respectively. Extracted data include posts by PHAs, comments from Facebook users, and their corresponding reactions, a feature in Facebook where users can interact with a Facebook status update, article, or a photo or video using one of six emotional reactions: Like, Love, Haha, Wow, Sad, and Angry. Contents posted between January 1, 2019, and March 18, 2020, were analyzed. Posts before January 1, 2020, were considered pre-COVID-19, and posts after January 1, 2020, were considered peri-COVID-19. We selected January 1, 2020, as the starting point for peri-COVID-19 since the statement on “Precautionary Measures in Response to Severe Pneumonia Cases in Wuhan, China,” was issued by the MOH on January 2, 2020 [33]. COVID-19-related posts were filtered out by manually scanning through the textual content of the posts.

### Data Analysis

#### Extent of Facebook Use

To examine the extent of Facebook use by the MOH, the CDC, and PHE, we calculated the average number of daily posts and compared pre-COVID-19 and peri-COVID-19 phases. To identify how specific events may influence the extent of Facebook use, we related the number of daily posts to the key dates on which the three countries reported their first COVID-19 cases or declared the outbreak as a national-level pandemic through the average number of posts per day (PPD) measure. To evaluate the extent of public engagement with the Facebook posts, we calculated the average number of reactions per post (RPP), average number of shares per post (SPP), and average number of comments per post (CPP) for pre- and peri-COVID-19 periods but focusing on COVID-19-related posts for the peri-COVID-19 period.

#### Thematic Analysis of COVID-19 Posts

The prevalent theme of each COVID-19 post was identified using a process involving two coders. First, the principal coder reviewed the contents of the COVID-19 posts by PHAs and assigned the relevant themes [34]. Next, another coder reviewed and confirmed the themes assigned by the first coder. The content of the posts were first screened through and condensed into short units. The predominant theme conveyed in the post (ie, the theme taking the larger proportion of the message) was assigned to posts with more than one theme. The list of themes included *situation update, preventive measures, appreciation, public reassurance, disease information, falsehood correction, face mask, research, testing and diagnosis, and miscellaneous*. For each of these themes, the number of constituent posts and percentage of these posts to the total number of posts were reported.

#### Sentiment and Emotion Analyses of Comments to COVID-19 Posts

To gain insights into the reactions and comments by Facebook users on the posts by PHAs, we conducted sentiment and emotion analyses on comments written in the English language. The sentiment polarity (SP) score for each comment was identified using the Vader algorithm [35] since the algorithm has been specifically conceptualized for ascertaining the sentiment in short texts (eg, user comments on Facebook or tweets from Twitter). The SP score value ranges from –1 to 1 and was classified into five categories: *very* *negative (–1<score<–0.5), negative (–0.5<score<0), neutral (score=0), positive (0<score<0.5), or very positive (0.5<score<1).*

For emotion analysis, we went beyond the emotions available as Facebook reactions. We adopted the eight emotions put forth in the theory of emotion [36] and classified the emotions of the comments as: *anger, fear, sadness, disgust, surprise, anticipation, trust,* or *joy*. Among the eight emotions, *trust* and *joy* are positive emotions, while *anger*, *sadness*, *fear,* and *disgust* are considered negative emotions. *Surprise* and *anticipation* can be either positive or negative depending on the context, hence not included in either of the two categories. The emotions conveyed in the comments were identified with the help of the DeepMoji algorithm [37] using a two-step process. First, the comments were analyzed using the DeepMoji algorithm, which recommended emojis based on the textual content. Second, the emotion that was mapped to the first-ranked emoji was considered as the emotion for the comment. The table in Multimedia Appendix 1 lists the mapping between the emojis and the corresponding emotions.

The total number of comments, CPP, SP scores, positive to negative sentiments ratio (PNSR) and positive to negative emotions ratio (PNER) were reported. PNSR and PNER are two valid measures that have been used in prior studies for sentiment and emotion analysis in texts [38-40]. The number of comments and the SP scores were plotted alongside the dates of key events.

#### Identification of Toxicity in Comments of COVID-19 Posts

Besides the general sentiment and emotion analyses, we identified toxic comments that may warrant intervention. Toxicity is defined as “the usage of rude, disrespectful, or unreasonable language that will likely provoke or make another user leave a discussion” [41]. In this study, the Perspective application program interface (API) service of Google [42] was used to measure the toxicity of comments. The toxicity score ranges between 0 (nontoxicity) and 1 (full toxicity). We further categorized the comments into a dichotomous variable, where comments with a toxicity score greater than or equal to 0.75 are toxic and comments with toxicity scores less than 0.75 are nontoxic. We examined the number of likes and replies per toxic comment to determine if such toxic comments may have any ripple effect. We compared this to the number of likes and replies per nontoxic comment as a reference.

## Results

### COVID-19 Outreach Efforts of PHAs and Public Engagement

**Figure 1:**
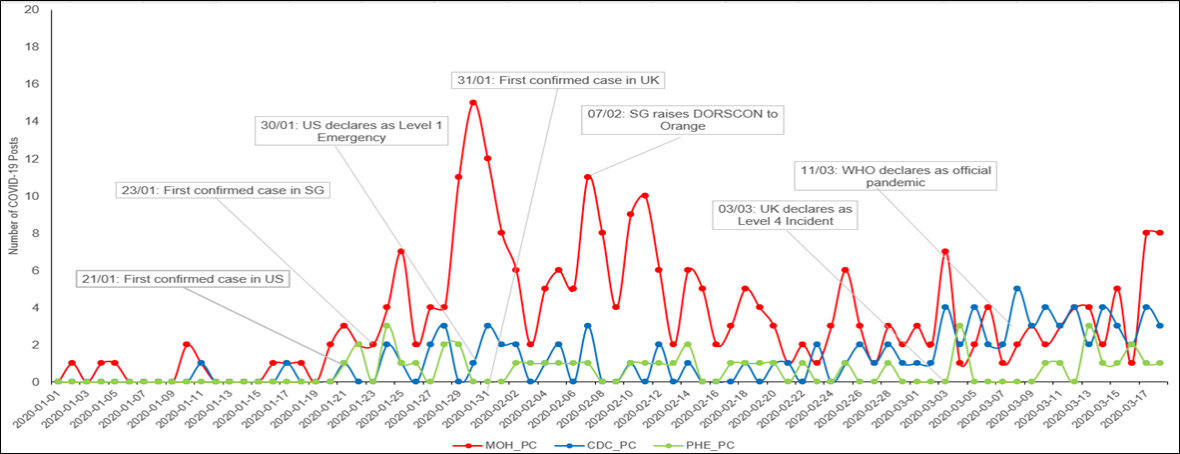
COVID-19 posts frequency during the analysis period. CDC_PC: Centers for Disease Control and Prevention's number of posts; COVID-19: coronavirus disease; DORSCON: Disease Outbreak Response System Condition; MOH_PC: Ministry of Health's number of posts; PHE_PC: Public Health England number of posts; SG: Singapore; WHO: World Health Organization.

In Table 1 and 2, the aggregated statistics related to the Facebook posts from the MOH, the CDC, and PHE are listed along with the public engagement metrics. In Figure 1, the COVID-19 posts daily count values are plotted in a line graph. In addition, the key dates on which the three countries reported their first COVID-19 cases and declared the outbreak as a national-level pandemic (in national terminology) are highlighted in the figure.

In the pre-COVID-19 phase (January 1, 2019, to December 31, 2019), the CDC had an average of 2 daily posts, while the MOH and PHE had an average of 1 daily post (Table 1). The MOH stepped up on the use of Facebook for public engagement during COVID-19. As of March 18, 2020, the MOH had published 304 posts peri-COVID-19 (January 1, 2020, to March 18, 2020), which was nearly a threefold increase in their PPD from 1.35 to 4.34. The MOH had the highest frequency of postings among the three PHAs with 304 posts, and the majority were related to COVID-19. The mean number of daily posts was 4.3, ranging from 1 to 15 posts daily. February was the most active month with 132 COVID-19 posts. Prior to the first locally confirmed case in Singapore on January 23, 2020, there was a limited number of daily posts from the MOH (ranging from 1 to 3 daily posts). However, the number of daily posts from the MOH increased to 7 two days after the first confirmed case in Singapore, and there have been at least 2 PPD ever since.

Of the total CDC posts, less than half were related to COVID-19. It published the highest number of COVID-19 posts in March, with 53 posts in the first 18 days of the month. The highest number of posts published on a single day was on March 8, 2020, with 5 posts. The CDC started to post at least 1 COVID-19 post every day starting on February 25, 2020. The number of daily posts remained at low levels even after the United States had declared a level 1 emergency on January 30, 2020. The average number of daily posts increased only after the United States declared COVID-19 as a level 1 emergency on January 30, 2020, and rose again after the WHO declared a pandemic on March 11, 2020.

PHE had the lowest posting frequency with only about half of posts being related to COVID-19 posts. Similar to the MOH, February was the most active month with 19 COVID-19 posts for PHE, and the highest number of posts on a single day (n=3) was done on 3 days: January 24, March 4, and March 13, 2020. Despite the United Kingdom declaring COVID-19 as a level 4 incident on March 3, 2020, there were days when PHE had zero COVID-19 posts. PHE started posting at least one COVID-19 post every day on March 13, 2020.

Compared to the pre-COVID-19 phase (Table 2), there was also a considerable increase in the public engagement metrics in the peri-COVID-19 time period. For instance, the MOH had a 7-fold increase in CPP, with a higher average number of people commenting in 2020 compared to 2019. The CDC saw a 9-fold increase in the mean RPP from 2019 to 2020 and close to a 10-fold increase in mean SPP from 2019 to 2020. In the case of PHE, the biggest rise is seen for SPP, with nearly a 5-fold increase from 2019 to 2020.

We have also noted that Facebook users who read peri-COVID-19 posts from the MOH were more likely to react to the post than to share or comment on the post, while Facebook users who read posts from the CDC and PHE were more likely to share the posts than to react or comment on the post. This observation was consistent in both pre- and peri-COVID-19 periods.

**Table 1:** Summary of COVID-19 Facebook outreach by the MOH, the CDC, and PHE during the periods of pre-COVID-19 (January 1, 2019, to December 31, 2019) and peri-COVID-19 (January 1, 2020, to March 18, 2020).

Agency	Outreach effort (pre-COVID-19^a^)		Outreach effort (peri-COVID-19)			
	Total posts, n	PPD^b^, mean (SD)	Total posts, n	PPD, mean (SD)	COVID-19 posts, n (%)	COVID-19 PPD Since first reported case in each country, mean (SD)
MOH^c^	192	1.4 (1.09)	304	4.3 (3.5)	271 (89.1)	5.0 (3.6)
CDC^d^	599	2.1 (0.99)	232	2.1 (1.1)	94 (40.5)	2.2 (1.1)
PHE^e^	346	1.3 (0.56)	87	1.6 (0.8)	45 (51.7)	1.4 (0.7)

^a^COVID-19: coronavirus disease.

^b^PPD: posts per day.

^c^MOH: Ministry of Health.

^d^CDC: Centers for Disease Control and Prevention.

^e^PHE: Public Health England.

**Table 2:** Summary of COVID-19 Facebook engagement by the MOH, the CDC, and PHE during the periods of pre-COVID-19 (January 1, 2019, to December 31, 2019) and peri-COVID-19 (January 1, 2020, to March 18, 2020).

Agency	Public engagement (pre-COVID-19^a^)			Public engagement (peri-COVID-19)		
	RPP^b^, mean (SD)	SPP^c^, mean (SD)	CPP^d^, mean (SD)	RPP_C19^e^, mean (SD)	SPP_C19^f^, mean (SD)	CPP_C19^g^, mean (SD)
MOH^h^	34.3 (26.3)	20.1 (100.3)	2.2 (4.5)	188.9 (201.4)	84.6 (279.7)	15.6 (20.7)
CDC^i^	230.7 (203.4)	240.9 (697.8)	43.1 (72.7)	2128.2 (4864.9)	2373.8 (3485.8)	255.3 (298.3)
PHE^j^	52.4 (68.7)	102.6 (212.5)	4.3 (6.7)	101.5 (89.8)	478.9 (568.1)	12.5 (11.5)

^a^COVID-19: coronavirus disease.

^b^RPP: reactions per post.

^c^SPP: shares per post.

^d^CPP: comments per post.

^e^RPP_C19: reactions to COVID-19 post.

^f^SPP_C19: shares per COVID-19 post.

^g^CPP_C19: comments per COVID-19 post.

^h^MOH: Ministry of Health.

^i^CDC: Centers for Disease Control and Prevention.

^j^PHE: Public Health England.

### Thematic Analysis

The themes *appreciation, research, testing and diagnosis,* and *miscellaneous* were combined to the theme *others* to focus on six major themes. As shown in Table 3, the themes from the MOH are more diverse, with no theme exceeding 30% of the total posts. In contrast, the CDC and PHE posts were largely related to *preventive measures*. For the MOH, *situation updat*e and *preventive measures* were the top two themes. Interestingly, the CDC and PHE did not issue any post to correct false information, while the MOH issued 16 of such posts. There was also no post from PHE and only 2 posts from the CDC to reassure their Facebook followers, while the MOH issued 32 of such *public reassurance* posts.

**Table 3:** Thematic analysis of the public health authorities’ coronavirus disease outreach efforts.

Theme	MOH^a^ (n=271), n (%)	CDC^b^ (n=94), n (%)	PHE^c^ (n=45), n (%)
Preventive measures	60 (22.1)	50 (53.2)	18 (40.0)
Situation update	78 (28.8)	21 (22.3)	7 (15.6)
Disease information	16 (5.9)	17 (18.1)	17 (37.8)
Public reassurance	32 (11.8)	2 (2.1)	0 (0.0)
Falsehood correction	16 (5.9)	0 (0.0)	0 (0.0)
Others	69 (25.5)	4 (4.3)	3 (6.7)

^a^MOH: Ministry of Health.

^b^CDC: Centers for Disease Control and Prevention.

^c^PHE: Public Health England.

### Sentiment and Emotion Analyses of Comments to COVID-19 Posts

The number of CPP was highest for the CDC, which was attributed to the high number of followers on their Facebook page (Table 4). Although the MOH had fewer number of followers than PHE, the number of comments received for the MOH’s COVID-19 posts were 5 times more than PHE. This observation can also be attributed to the high number of COVID-19 posts for the MOH vs PHE’s COVID-19 posts. Interestingly, the mean CPP of PHE was still higher than the MOH. The average SP scores of all three PHAs were close to the neutral sentiment mark of zero with only the MOH being slightly positive. Correspondingly, the PNSR and PNER of the MOH were much higher than the values for the CDC and PHE. However, since all these values were below 1, it is an indication that there were more negative sentiments and emotions conveyed in the comments. The CDC and PHE received predominantly negative comments from their followers based on the low PNSR and PNER values.

**Figure 2:**
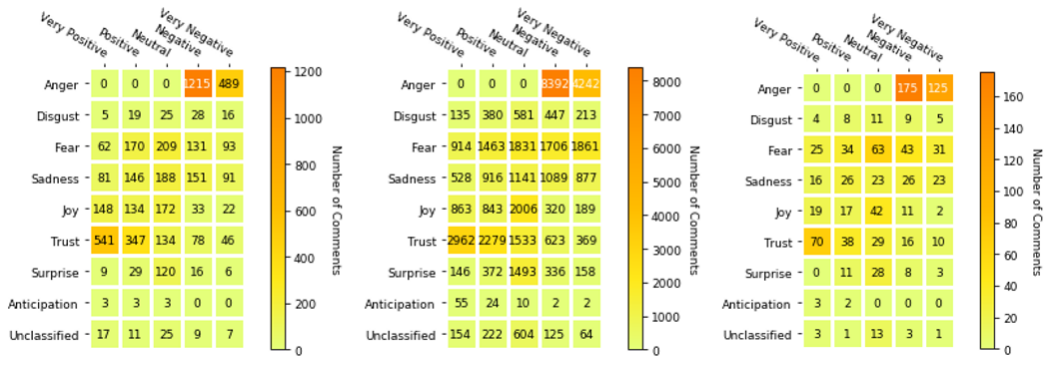
Sentiment and emotions heat map for coronavirus disease Facebook comments (left: Ministry of Health; middle: Centers for Disease Control and Prevention; right: Public Health England).

In Figure 2, the emotion categories are plotted against the sentiment categories, with a darker shade of the box reflecting a higher number of comments. We observed that most of the comments to the MOH posts were angry (n=1704, 33.9%), with 1215 being angry and negative and 489 being angry and very negative. Anger is also the most prevalent emotion for the CDC (n=12,634/42,470, 29.8%) and PHE (n=300/977, 30.7%) posts. For the MOH, the negative emotions (anger, disgust, fear, and sadness) account for 62.0% (n=3119/5032) of the comments, while positive emotions (trust and joy) accounted for 33.0% (n=1655) of the comments. In the case of the CDC and PHE, negative emotions accounted for 63.0% (n=26,716/42,470) and 66.2% (n=647/977) of the comments, respectively, and positive emotions accounted for 28.2% (n=11,987) and 26.0% (n=254) of comments, respectively. Since emotions have a direct effect on sentiments, the negative sentiments accounted for the majority of the comments (n=2431/5032, 48.3%; n=21,015/42,470, 49.5%; and n=491/977, 50.3% for the MOH, the CDC, and PHE, respectively). The MOH had a higher percentage of positive sentiments (n=1725/5032, 34.3%) compared to the CDC (n=12,256/42,470, 28.9%) and PHE (n=277/977, 28.4%).

**Figure 3:**
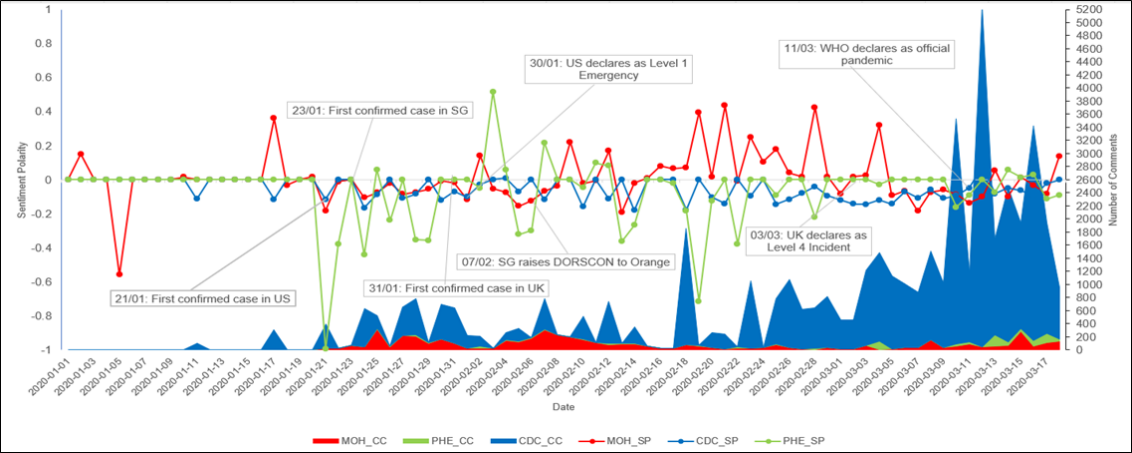
Temporal trend analysis for the number of comments and sentiment polarity. CDC_CC: Centers for Disease Control and Prevention's number of comments; CDC_SP: Centers for Disease Control and Prevention's sentiment polarity score; DORSCON: Disease Outbreak Response System Condition; MOH_CC: Ministry of Health's number of comments; MOH_SP: Ministry of Health's sentiment polarity score; PHE_CC: Pubilc Health England's number of comments; PHE_SP: Public Health England's sentiment polarity score; SG: Singapore; WHO: World Health Organization.

The temporal trend analysis (Figure 3) provides more information compared to the snapshots provided in Table 4 and Figure 2. For instance, we observed that the number of comments increased significantly over time for the CDC posts, while the number of comments for the MOH posts appeared to have decreased over time. Among the three agencies, PHE had the highest degree of fluctuations in SP scores, with many negative and few positive spikes. The SP scores were mostly negative for the CDC posts, while SP scores were positive for the MOH on several occasions, which contributed to an average SP that tends toward neutral. The MOH had the highest number of days with positive sentiments, particularly the period between February 16, 2020, and March 5, 2020, which could be due to a relatively high number of appreciation posts (n=9) during that period.

**Table 4:** Emotion and sentiment analyses of COVID-19 Facebook comments.

Agency	COVID-19^a^ posts, n	Comments, n	CPP^b^, mean (SD)	SP^c^, mean (SD)	PNSR^d^, mean (SD)	PNER^e^, mean (SD)
MOH^f^	271	5032	18.57 (30.04)	0.02 (0.25)	0.94 (2.11)	0.84 (1.79)
CDC^g^	94	42,470	451.81 (529.09)	–0.09 (0.06)	0.57 (0.16)	0.41 (0.12)
PHE^h^	45	977	21.71 (31.89)	–0.14 (0.26)	0.55 (0.49)	0.44 (0.54)

^a^COVID-19: coronavirus disease.

^b^CPP: comments per post.

^c^SP: sentiment polarity score.

^d^PNSR: positive to negative sentiments ratio.

^e^PNER: positive to negative emotions ratio.

^f^MOH: Ministry of Health.

^g^CDC: Centers for Disease Control and Prevention.

^h^PHE: Public Health England.

### Identification of Toxicity in Comments of COVID-19 Posts

The CDC had the highest number of toxic comments, followed by the MOH and PHE (Table 5). PHE had the highest average of likes per toxic comment (LPTC) compared to the CDC and the MOH. For the PHE page, toxic comments received more likes and replies from its followers as compared to nontoxic comments. The mean LPTC for PHE was two times higher compared to nontoxic comments. Similarly, toxic comments received more replies compared to nontoxic comments.

**Table 5:** Summary of coronavirus disease toxic comments.

Agency	Total comments, n	Toxic comments, n (%)	LPTC^a^, mean	LPNC^b^, mean	RPTC^c^, mean	RPNC^d^, mean
MOH^e^	5032	58 (0.01)	0.47	1.38	0.14	0.43
CDC^f^	42,470	728 (0.01)	0.89	1.99	0.13	0.71
PHE^g^	977	12 (0.01)	2.42	1.44	0.92	0.60

^a^LPTC: likes per toxic comment.

^b^LPNC: likes per nontoxic comment.

^c^RPTC: replies per toxic comment.

^d^RPNC: replies per nontoxic comment.

^e^MOH: Ministry of Health.

^f^CDC: Centers for Disease Control and Prevention.

^g^PHE: Public Health England.

## Discussion

### Principal Findings

Among the three agencies, the MOH was the most active in using Facebook to reach out to its followers in terms of posting frequency, with an average of around 4 posts daily, exceeding both the CDC and PHE. The MOH displayed a similar active outreach strategy during the previous Zika outbreak in 2016 by ramping up engagement online with more frequent posting [21]. The MOH COVID-19 posts received more attention from their followers compared to the previous outbreak. For instance, the average number of comments received per post was 3.6 for Zika posts [21], while for COVID-19 posts, the average has increased to 15.6.

The posting frequency of the CDC and PHE on matters pertaining to COVID-19 was low in the initial peri-COVID-19 phase; this was because a substantial number of posts were still dedicated to other public health topics (eg, mental health, food disease outbreak, chronic disease management). This may reflect that both countries perceived the risk to be low or possibly that the outbreak was still largely confined within Asia. At the time of analysis, the epicenter of the COVID-19 pandemic was Wuhan, China with several other Asian countries, including Singapore. We observed that the volume of updates on COVID-19 related to prevention from the CDC and PHE increased toward the end of the analysis period, which parallels the surge in case count in both the United States and England. Given the large number of followers on the CDC and PHE Facebook pages, it is a missed opportunity that the CDC and PHE did not engage with their followers more intensively using Facebook. We observed that Facebook users who engaged with the CDC or PHE posts were more likely to share rather than to react or comment on the posts. Hence, Facebook may possibly be a useful platform for the CDC or PHE to disseminate information for Facebook users to propagate to others.

In our analysis of the PHAs’ post content, we restricted the number of themes to six to focus on major themes, unlike earlier studies where more themes were used with a meagre number of posts for certain themes [26]. The posts from the MOH were more diverse with frequent updates on preventive measures, travel advisories, disease information, falsehood correction, and even appreciation for health care workers and other frontline staff. This was in contrast to the posts from the CDC and PHE, where the messages were mostly focused on preventive measures and sporadic situation updates. Falsehood corrections are in need during this pandemic, as an earlier study identified that misleading Facebook posts acquired more popularity than accurate posts during the Zika outbreak in the United States [43]. On this point, we observed that the MOH has adopted misinformation debunking as one of its community and social measures for handling the COVID-19 situation [6]. The current distribution of themes may reflect the different phases that the three countries were going through during the time of our analyses. We anticipate that as the pandemic develops across each country the themes of the posts will continually evolve. Nevertheless, there is a need to enhance awareness and not undermine the possibility of a serious outbreak during the precrisis period [22]. From our analyses, we did not identify much evidence to show attempts at such efforts from the CDC and PHE in their official Facebook pages.

In previous disease pandemics, negative sentiments were generally prevalent in social media [23]. We made similar findings in our analyses where the majority of the posts conveyed anger emotions and negative sentiments. For the MOH, however, we noticed that over time (from mid-February to the first week of March 2020), Facebook users began to be more positive about the government’s response to the pandemic. This demonstrates that monitoring sentiments and emotions on social media can help PHAs gauge the effectiveness of their public health education efforts on Facebook. Another observation that supports the monitoring of sentiments and emotions on social media is that the number of comments tend to spike in conjunction with specific events. For example, our data showed that the number of comments sharply rose in association with the first confirmed case in the United States (January 21, 2020), when Singapore rose its DORSCON level to orange (February 7, 2020), when the United Kingdom declared COVID-19 as a Level 4 incident (March 3, 2020), and when the WHO declared COVID-19 a pandemic (March 11, 2020).

The prevalence of toxic comments for all three Facebook pages’ COVID-19 posts was fairly low. It is possible that the majority of the toxic comments had been removed (for instance, the CDC has a policy that profane and obscene comments can be deleted [44]), and what was analyzed were those that were not filtered. The US President has repeatedly referred to SARS coronavirus 2 as the “Chinese virus,” and this may have led to anti-Asian sentiments [45]. This may explain why the CDC had the highest number of toxic comments. Fortunately, the volume of such comments remained low, and the agreement with such toxic comments was also low as reflected by the low average number of likes. PHAs should consider dedicating resources during a pandemic to manage toxic comments as well as combat falsehood. We observed that both the CDC and PHE did not have any post to correct falsehoods, unlike the MOH. In contrast, social media platforms have been proactive in setting up centralized hubs dedicated to COVID-19 updates and information such as the COVID-19 Information Center on Facebook [46] or the COVID-19 Information and Resources collection on Google [47] to direct social media users to trusted and reliable information.

### Limitations

In this study, we analyzed data from Facebook only. However, PHAs may have used other social media platforms such as Twitter and YouTube to disseminate public health information to their citizens. Thus, this study’s findings may not fully represent the overall social media outreach efforts of PHAs during the COVID-19 pandemic. In addition, the sentiments captured on Facebook comments may not reflect the users of other social media platforms, as the user profiles of these various platforms are known to be different. Furthermore, PHAs may currently still use traditional news and mass media channels to reach the public with information, updates, and guidance measures. Hence, PHAs’ outreach efforts in social media platforms is supplementary, and these platforms are considered either as a resource for additional information or for reaching out to people who no longer follow traditional news and mass media channels. Another limitation of the study was that we have limited our analyses of the Facebook followers’ response to posts initiated by the PHAs. We have not, for instance, analyzed the comments within private circles or closed groups that may be different in nature compared to publicly disclosed comments. Our paper has focused on the comparative analysis of how three PHAs have used Facebook for COVID-19 communications strategy. We did not evaluate if the PHAs’ use of social media has improved over time as they gain experience from dealing with other infectious diseases such as the H1N1 swine flu pandemic, Ebola epidemic, or Zika outbreak. As social media platforms become more prominent, its users and the interactions among its users evolve. Optimal use of these platforms for public health communications will benefit from constant reflection and critical appraisal of what strategies have worked and what have not. Finally, the thematic analysis may be more robust if independent coding of the posts was conducted. However, the huge number of posts rendered this process time-consuming. Accordingly, the review and confirmation of the themes from a second coder was sought as an acceptable compromise.

### Conclusions

The Facebook postings by the PHAs in this study provided some insights into their governments’ COVID-19 broader communication strategy. Through our study, we identified differences in the Facebook-based outreach and engagement efforts of three developed countries during the prepandemic and peripandemic periods of COVID-19. The differences were found in terms of both posting frequency and themes in posts. The change in sentiments in response to specific outreach events were also observed. On the whole, the MOH stepped up its outreach efforts on Facebook more intensively compared to the CDC and PHE. We hope that our findings will be of interest to PHAs and health science researchers who study pandemics in the context of social media. In our upcoming work, we intend to conduct studies in two related directions. In our first set of studies, we intend to collect more data from the same three PHA Facebook pages and analyze the results with data segregated in three phases (pre-COVID-19, peri-COVID-19, and post-COVID-19). In the second set of studies, we intend to analyze the outreach efforts of other countries during this COVID-19 pandemic to understand the effectiveness and shortfalls of strategies used by different countries.

## Appendix

Multimedia Appendix 1 Emojis and the associated emotions.

## Abbreviations

API: application program interface
CDC: Centers for Disease Control and Prevention
COVID-19: coronavirus disease
CPP: comments per post
DORSCON: Disease Outbreak Response System Condition
LPTC: likes per toxic comment
MOH: Ministry of Health
PHA: public health authority
PHE: Public Health England
PNER: positive to negative emotions ratio
PNSR: positive to negative sentiment ratio
PPD: posts per day
RPP: reactions per post
SARS: severe acute respiratory syndrome
SP: sentiment polarity
SPP: shares per post
WHO: World Health Organization
