# Supplementary material for: Measuring the Outreach Efforts of Public Health Authorities and the Public Response on Facebook During the COVID-19 Pandemic in Early 2020: Cross-Country Comparison
Source: J Med Internet Res. 2020 May 19;22(5):e19334. doi: 10.2196/19334 (PMC7238862; doi:10.2196/19334)
Supplement: Multimedia Appendix 1 [file jmir_v22i5e19334_app1.docx]

**Table A1.** Emojis and the associated emotions

| Emoji Code | Emotion |
| --- | --- |
| :joy: | Joy |
| :unamused: | Disgust |
| :weary: | Sadness |
| :sob: | Sadness |
| :heart_eyes: | Joy |
| :pensive: | Sadness |
| :ok_hand: | Trust |
| :blush: | Joy |
| :heart: | Anticipation |
| :smirk: | Joy |
| :grin: | Surprise |
| :notes: | Unclassified |
| :flushed: | Surprise |
| :100: | Trust |
| :sleeping: | Unclassified |
| :relieved: | Joy |
| :relaxed: | Joy |
| :raised_hands: | Joy |
| :two_hearts: | Joy |
| :expressionless: | Anger |
| :sweat_smile: | Fear |
| :pray: | Trust |
| :confused: | Sadness |
| :kissing_heart: | Joy |
| :heartbeat: | Joy |
| :neutral_face: | Sadness |
| :information_desk_person: | Disgust |
| :disappointed: | Sadness |
| :see_no_evil: | Disgust |
| :tired_face: | Disgust |
| :v: | Trust |
| :sunglasses: | Joy |
| :rage: | Anger |
| :thumbsup: | Trust |
| :cry: | Sadness |
| :sleepy: | Sadness |
| :yum: | Joy |
| :triumph: | Anger |
| :hand: | Disgust |
| :mask: | Fear |
| :clap: | Trust |
| :eyes: | Surprise |
| :gun: | Fear |
| :persevere: | Sadness |
| :smiling_imp: | Joy |
| :sweat: | Fear |
| :broken_heart: | Sadness |
| :yellow_heart: | Trust |
| :musical_note: | Unclassified |
| :speak_no_evil: | Fear |
| :wink: | Joy |
| :skull: | Fear |
| :confounded: | Sadness |
| :smile: | Joy |
| :stuck_out_tongue_winking_eye: | Joy |
| :angry: | Anger |
| :no_good: | Disgust |
| :muscle: | Joy |
| :facepunch: | Trust |
| :purple_heart: | Unclassified |
| :sparkling_heart: | Joy |
| :blue_heart: | Unclassified |
| :grimacing: | Fear |
| :sparkles: | Joy |
